# Multicenter Evaluation of the BIOFIRE Blood Culture Identification 2 Panel for Detection of Bacteria, Yeasts, and Antimicrobial Resistance Genes in Positive Blood Culture Samples

**DOI:** 10.1128/jcm.01891-22

**Published:** 2023-05-25

**Authors:** Daniel D. Rhoads, Spyros Pournaras, Amy Leber, Joan-Miquel Balada-Llasat, Amanda Harrington, Vittorio Sambri, Rosemary She, Gregory J. Berry, Judy Daly, Caryn Good, Aikaterini Tarpatzi, Kathy Everhart, Tai Henry, Kathleen McKinley, Silvia Zannoli, Pil Pak, Fan Zhang, Rebecca Barr, Kristen Holmberg, Bart Kensinger, Daisy Y. Lu

**Affiliations:** a Cleveland Clinic Lerner College of Medicine, Case Western Reserve University, Cleveland, Ohio, USA; b Department of Laboratory Medicine, Cleveland Clinic, Cleveland, Ohio, USA; c Infection Biology Program, Lerner Research Institute, Cleveland Clinic, Cleveland, Ohio, USA; d Laboratory of Clinical Microbiology, Attikon University Hospital, School of Medicine, National and Kapodistrian University of Athens, Athens, Greece; e Nationwide Children’s Hospital, Columbus, Ohio, USA; f The Ohio State University Wexner Medical Center, Columbus, Ohio, USA; g Loyola University Medical Center, Maywood, Illinois, USA; h The Greater Romagna Area Hub Laboratory, Cesena, Italy; i DIMES, University of Bologna, Bologna, Italy; j Keck School of Medicine of University of Southern California, Los Angeles, California, USA; k Northwell Health Laboratories, Lake Success, New York, USA; l Primary Children’s Hospital, Salt Lake City, Utah, USA; m University Hospitals Cleveland Medical Center, Cleveland, Ohio, USA; n bioMérieux, Inc., Salt Lake City, Utah, USA; Medical College of Wisconsin

**Keywords:** BCID2, PCR, antimicrobial resistance, bacteremia, blood culture, rapid diagnostics, sepsis

## Abstract

Diagnostic tools that can rapidly identify and characterize microbes growing in blood cultures are important components of clinical microbiology practice because they help to provide timely information that can be used to optimize patient management. This publication describes the bioMérieux BIOFIRE Blood Culture Identification 2 (BCID2) Panel clinical study that was submitted to the U.S. Food & Drug Administration. Results obtained with the BIOFIRE BCID2 Panel were compared to standard-of-care (SoC) results, sequencing results, PCR results, and reference laboratory antimicrobial susceptibility testing results to evaluate the accuracy of its performance. Results for 1,093 retrospectively and prospectively collected positive blood culture samples were initially enrolled, and 1,074 samples met the study criteria and were included in the final analyses. The BIOFIRE BCID2 Panel demonstrated an overall sensitivity of 98.9% (1,712/1,731) and an overall specificity of 99.6% (33,592/33,711) for Gram-positive bacteria, Gram-negative bacteria and yeast targets which the panel is designed to detect. One hundred eighteen off-panel organisms, which the BIOFIRE BCID2 Panel is not designed to detect, were identified by SoC in 10.6% (114/1,074) of samples. The BIOFIRE BCID2 Panel also demonstrated an overall positive percent agreement (PPA) of 97.9% (325/332) and an overall negative percent agreement (NPA) of 99.9% (2,465/2,767) for antimicrobial resistance determinants which the panel is designed to detect. The presence or absence of resistance markers in *Enterobacterales* correlated closely with phenotypic susceptibility and resistance. We conclude that the BIOFIRE BCID2 Panel produced accurate results in this clinical trial.

## INTRODUCTION

Bloodstream infections are associated with high morbidity and mortality, and the rapidity of detection, characterization, and chemotherapeutic management of these microbes impacts the clinical outcome of patients with bacteremia and fungemia. Therefore, the discipline of clinical microbiology continues to focus on developing tools that can more rapidly detect and characterize microbes causing bloodstream infections. The BIOFIRE Blood Culture Identification 2 (BCID2) Panel is an *in vitro* diagnostic device that uses nucleic acid amplification testing to detect and identify common pathogens and contaminants isolated from blood cultures.

Standard-of-care (SoC) detection and reference standard detection of bacteremia and fungemia are currently based on broth blood cultures of the microbes from blood specimens. Blood specimens are collected directly into broth bottles and then incubated upon receipt in the laboratory using a continuously monitored blood culture instrument, which identifies microbial presence in the bottles by detecting metabolic by-products produced by the microbes during their growth and replication. When microbial presence is detected by the instrument, a portion of the positive blood culture (PBC) is examined microscopically and subcultured to nutrient agar media. Additionally, rapid tests such as the BIOFIRE BCID2 Panel can be performed at the time microbial growth is detected in the PBC. These rapid tests have the greatest clinical impact and financial value when employed in close partnership with caregivers outside the laboratory who are ready to quickly act on these rapid test results (1, 2).

The current study describes the prospective clinical evaluation of the BIOFIRE BCID2 Panel that was submitted to the U.S. Food and Drug Administration (FDA) for clearance as an *in vitro* diagnostic test. The BIOFIRE BCID2 Panel is a second-generation assay, which is an iterative improvement of the BIOFIRE Blood Culture Identification (BCID) Panel (3, 4). The BIOFIRE BCID2 Panel is differentiated from the original BIOFIRE BCID Panel by increased specificity of some taxonomic identification, additional identifiable taxa, and additional genetic resistance markers (Table 1).

**TABLE 1:** Analytes detected by the BIOFIRE BCID2 Panel

Category	Analyte^*a*^
Gram-positive bacteria	**Enterococcus faecalis** ^*b*^
	**Enterococcus faecium** ^*b*^
	Listeria monocytogenes
	**Staphylococcus spp.**
	Staphylococcus aureus
	**Staphylococcus epidermidis**^*b*^
	**Staphylococcus lugdunensis**^*b*^
	**Streptococcus spp.**
	Streptococcus agalactiae (group B)
	Streptococcus pneumoniae
	Streptococcus pyogenes (group A)

Gram-negative bacteria	**Acinetobacter calcoaceticus**-**A. baumannii complex**^*c*^
	**Bacteroides fragilis** ^*d*^
	Haemophilus influenzae
	Neisseria meningitidis
	Pseudomonas aeruginosa
	**Stenotrophomonas maltophilia** ^*d*^
	**Enterobacterales**
	Enterobacter cloacae complex^*e*^
	Escherichia coli
	**Klebsiella aerogenes**^*b*^
	Klebsiella oxytoca
	Klebsiella pneumoniae group^*f*^
	Proteus spp.
	**Salmonella spp.**^*b*^
	Serratia marcescens

Yeasts	Candida albicans
	**Candida auris** ^*d*^
	Candida glabrata
	Candida krusei
	Candida parapsilosis
	Candida tropicalis
	**Cryptococcus neoformans**/**C. gattii**^*d*^

AMR genes	**CTX-M** ^*d*^
	**IMP** ^*d*^
	KPC
	***mcr-1*** ^*d*^
	*mecA*/*C*
	***mecA*/*C* and MREJ (MRSA)** ^*d*^
	**NDM** ^*d*^
	**OXA-48-like** ^*d*^
	*vanA*/*B*
	**VIM** ^*d*^

a Analyte new to the BIOFIRE BCID2 Panel or modified from the BIOFIRE BCID Panel are in boldface.

b Species/genus-level interpretation in place of the higher-level interpretation in the original BIOFIRE BCID Panel. Analytes new to the BIOFIRE BCID2 Panel or modified from the BIOFIRE BCID Panel are in boldface.

c Analytical testing and/or sequence analysis demonstrated reactivity with the Acinetobacter calcoaceticus*-*A. baumannii complex species: Acinetobacter baumannii, Acinetobacter calcoaceticus, Acinetobacter dijkshoorniae (synonymous with Acinetobacter lactucae), Acinetobacter nosocomialis, Acinetobacter pittii, and Acinetobacter seifertii.

d Analyte new to the BIOFIRE BCID2 Panel. Analytes new to the BIOFIRE BCID2 Panel or modified from the BIOFIRE BCID Panel are in boldface.

e Analytical testing and/or sequence analysis demonstrated reactivity with Enterobacter cloacae, Enterobacter asburiae, Enterobacter hormaechei, Enterobacter kobei, Enterobacter ludwigii, and Enterobacter mori.

f Analytical testing and/or sequence analysis demonstrated reactivity with Klebsiella pneumoniae (KPI), Klebsiella quasipneumoniae (KPII), and Klebsiella variicola (KPIII).

## MATERIALS AND METHODS

### Study overview.

The study was conducted at nine sites across the United States and Europe over a period of approximately 8 months (October 2018 to May 2019). At each study site, a waiver of the requirement for informed consent was obtained from the Institutional Review Board (IRB) for the use of residual blood culture samples created during SoC.

Aerobically and anaerobically cultured samples identified as positive for microbial growth by an automated continuous monitoring blood culture system (CMBCS) were frozen or tested using the BIOFIRE BCID2 Panel within 24 h of positivity. PBC samples collected from October 2018 through February 2019 were immediately frozen for future testing, and fresh PBC samples were collected between January and May 2019. Frozen samples were tested immediately after thawing, and fresh samples were tested within 24 h of positive blood bottle indication. All samples were tested between January 2019 and May 2019. Sample enrollment was not discriminated based on subject age, sex, patient location, or diagnosis; however, PBC bottles containing charcoal in the culture medium were excluded, which is in agreement with the FDA-cleared instructions for use, and only one sample per subject was used in the study.

SoC subculture and isolate identification were performed per each laboratory’s routine practice, and this identification was used as the comparator method for organism identification. The comparator method for antimicrobial resistance (AMR) genes was not obtained using each laboratory’s SoC testing. Instead, AMR genes were identified by comparing to FDA-cleared predicate devices or PCR assays performed in a research setting.

### BIOFIRE BCID2 Panel testing.

An investigational-use-only (IUO) version of the BIOFIRE BCID2 Panel that is identical to the commercial (i.e., FDA-cleared [5], CE-marked) *in vitro* diagnostic (IVD) version was used in this study. All specimen manipulation was performed with good laboratory biosafety practices and in accordance with the manufacturer’s instructions (https://www.online-ifu.com/ITI0048). The BIOFIRE BCID2 Panel incorporates internal controls, automated nucleic acid extraction, amplification, detection, and qualitative result interpretation with a sample-to-answer time of approximately 1 h. The BIOFIRE BCID2 Panel simultaneously tests for 43 targets (Table 1), 18 of which are modified or new to the BIOFIRE BCID2 Panel compared to the BIOFIRE BCID Panel. The detection or lack of detection of each bacterial AMR gene is reported only if a potential microbial carrier of the gene is also detected (Table 2). Both FilmArray 2.0 and FilmArray Torch instruments were used with the BIOFIRE BCID2 Panel in the study. Quality control testing was performed each day of testing at each site. Environmental swab wipe tests were performed regularly on the BIOFIRE BCID2 Panel to ensure the absence of amplicon contamination near the testing location.

**TABLE 2:** AMR genes and applicable bacteria

BIOFIRE BCID2 Panel Result	AMR Gene(s)^*a*^									
	*vanA*/*B*	*mecA*/*C* and MREJ (MRSA)^*b*^	*mecA*/*C*	*mcr-1* ^*b*^	CTX-M^*b*^	IMP^*b*^	KPC	NDM^*b*^	OXA-48-like^*b*^	VIM^*b*^
Enterococcus faecalis	×									
Enterococcus faecium	×								
Staphylococcus aureus		×								
Staphylococcus epidermidis			×							
Staphylococcus lugdunensis			×							
Acinetobacter calcoaceticus-baumannii complex					×	×	×	×		×
*Enterobacterales*					×	×	×	×	×	×
Enterobacter cloacae complex				×	×	×	×	×	×	×
Escherichia coli				×	×	×	×	×	×	×
Klebsiella aerogenes				×	×	×	×	×	×	×
Klebsiella oxytoca				×	×	×	×	×	×	×
Klebsiella pneumoniae group				×	×	×	×	×	×	×
Proteus spp.					×	×	×	×	×	×
Salmonella spp.				×	×	×	×	×	×	×
Serratia marcescens					×	×	×	×	×	×
Pseudomonas aeruginosa					×	×	×	×		×

a If an applicable bacterium is not detected by the BIOFIRE BCID2 Panel, the AMR gene result is reported as “Not Applicable.”

b AMR analytes new to the BIOFIRE BCID2 Panel or modified from the BIOFIRE BCID Panel.

### Comparator testing.

**(i) Organism identification.** SoC methods to identify bacteria and fungi isolates from PBC were followed at each study site. A summary of each site’s SoC culture and organism identification methods is provided in Table 3. Discrepancies in identification between the BIOFIRE BCID2 Panel and SoC were investigated further. Additionally, all *Candida* species were further characterized by bidirectional sequencing to confirm the SoC identification.

**TABLE 3:** Summary of SoC methods used by each prospective site

Site no.	Blood culture bottle types	Blood culture system	MALDI-TOF^*a*^ brand and model for isolate ID
1	BD Bactec Plus Aerobic/F, BD Bactec Standard 10 Aerobic/F,BD Bactec Lytic/10 Anaerobic/F, BD Bactec Standard Anaerobic/F	BD Bactec FX	bioMérieux Vitek MS
2	BD Bactec Plus Aerobic/F, BD Bactec Lytic/10 Anaerobic/F, BD Bactec Plus Anaerobic/F, BD Bactec Peds Plus/F	BD Bactec FX	Bruker MALDI Biotyper
3	BD Bactec Plus Aerobic/F, BD Bactec Plus Anaerobic/F, BD Bactec Peds Plus/F	BD Bactec FX	Bruker Microflex LT
4	bioMérieux BacT/Alert FA Plus, bioMérieux BacT/Alert FN Plus, bioMérieux BacT/Alert PF Plus	bioMérieux BacT/Alert Virtuo	bioMérieux Vitek MS
5	bioMérieux BacT/Alert FA Plus, bioMérieux BacT/Alert SA, bioMérieux BacT/Alert SN	bioMérieux BacT/Alert Virtuo	Bruker MALDI Biotyper
6	BD Bactec Plus Aerobic/F, BD Bactec Lytic/10 Anaerobic/F	BD Bactec FX	Bruker MALDI Biotyper
7	bioMérieux BacT/Alert FA Plus, bioMérieux BacT/Alert SN	bioMérieux BacT/Alert3D	bioMérieux Vitek MS
8	bioMérieux BacT/Alert FA Plus, bioMérieux BacT/Alert FN Plus, bioMérieux BacT/Alert PF Plus	bioMérieux BacT/Alert Virtuo	bioMérieux Vitek MS
9	BD Bactec Plus Aerobic/F, BD Bactec Lytic/10 Anaerobic/F	BD Bactec FX	Bruker MALDI Biotyper

a MALDI-TOF, matrix-assisted laser desorption ionization–time of flight.

**(ii) AMR gene detection.** *(a) BIOFIRE BCID Panel.* All samples were interrogated for resistance markers using the BIOFIRE BCID2 Panel’s predicate device, which is the FDA-cleared BIOFIRE BCID Panel (BIOFIRE, Salt Lake City, UT). The BIOFIRE BCID Panel was the comparator test used for *mecA*/*C*, *vanA*/*B*, and KPC.

*(b) PCR assays.* AMR targets not included on the BIOFIRE BCID Panel (CTX-M, IMP, NDM, OXA-48-like, VIM, and *mcr-1*) were interrogated using single, nested PCR assays followed by bidirectional sequencing of amplicons. When feasible, the comparator PCR assays were designed to target different regions of the AMR genes than are targeted by the BIOFIRE BCID2 Panel. Comparator PCR assays were designed to generate sufficient sequence information for conclusive AMR gene identification. The comparator sequences for CTX-M, IMP, NDM, VIM and *mcr-1* were all downstream of the BIOFIRE BCID2 Panel target; most sequences contained some overlap between the panel target and the comparator target, but the *mcr-1* comparator region did not overlap. OXA-48-like used two comparator sequences: one was upstream with no overlap with the BIOFIRE BCID2 Panel target, and one was downstream with significant overlap.

*(c) Cepheid Xpert MRSA*/*SA BC.* The FDA-cleared Cepheid Xpert MRSA/SA BC test (Cepheid; Sunnyvale, CA) was used according to the product instructions for use (IFU) as the comparator for the BIOFIRE BCID2 Panel *mecA*/*C* and MREJ (methicillin-resistant Staphylococcus aureus [MRSA]) interpretations for Staphylococcus aureus. PBC samples that contained S. aureus (by either the BIOFIRE BCID2 Panel or SoC identification methods) were frozen and sent to a central lab and tested with the Xpert MRSA/SA BC test on the Cepheid GeneXpert I platform.

**(iii) ESBL phenotypic detection and antimicrobial susceptibility test interpretations.** Phenotypic detection of ESBL and susceptibility to cefepime, piperacillin-tazobactam, and meropenem were determined for all *Enterobacterales* isolates using broth microdilution (BMD) antimicrobial susceptibility testing. Custom-designed frozen BMD Sensititre trays (Thermo Fisher, Waltham, MA) were used for MIC testing of the isolates. Phenotypic detection of extended-spectrum β-lactamases (ESBL) was identified if clavulanic acid decreased the MIC of ceftazidime or cefotaxime by more than two doubling dilutions.

The Clinical and Laboratory Standards Institute (CLSI) document *Performance Standards for Antimicrobial Susceptibility Testing* (6) was used to interpret MIC results. *Enterobacterales* isolates with an MIC of ≤2 μg/mL of cefepime, ≤8/4 μg/mL of piperacillin-tazobactam, and ≤1 μg/mL of meropenem were interpreted as susceptible. *Enterobacterales* isolates with MICs of 4 to 8 μg/mL of cefepime and 16/4 μg/mL of piperacillin-tazobactam were interpreted as susceptible—dose dependent (SDD).

### Results and discrepancy analysis.

A BIOFIRE BCID2 Panel result was considered a true positive (TP) or true negative (TN) when it agreed with the result from the comparator method. A result was considered a false positive (FP) or false negative (FN) when it disagreed with the result from the comparator method. Sensitivity or positive percent agreement (PPA) was calculated as 100 × TP/(TP + FN), while specificity or negative percent agreement (NPA) was calculated as 100 × TN/(TN + FP). When sufficient PBC specimen volume and/or the isolate was available, discordant results were investigated using additional, independent molecular testing. Note that the performance data for sensitivity or PPA and specificity or NPA presented here consist of unresolved data, as presented in the IFU for the FDA-cleared test; discrepancy investigation is provided but was not used to recalculate performance data.

### Statistical analysis.

The exact binomial two-sided 95% confidence intervals (95% CI) were calculated for performance measures according to the Wilson score method (7).

## RESULTS

A single external control was successfully performed each day of testing at each of the nine testing sites. Each site rotated through one negative and four positive controls in succession. If a successful external control result was not obtained, all BIOFIRE BCID2 Panel runs from the site on that day were excluded from analysis. A total of 146 swab wipe tests were performed on the BIOFIRE BCID2 Panel to monitor the laboratory environment for possible contamination, and all results were reported as “not detected” for all reportable targets.

In total, 1,093 clinical samples were enrolled in the intent-to-test group. Of the 1,093 samples, 17 (1.5%) were excluded for not meeting the defined inclusion criteria, such as being tested more than 24 h after being flagged as positive or being a second sample from the same subject. One additional sample (0.1%) was excluded due to protocol deviation, and one additional sample (0.1%) was excluded because successful external control results were not obtained. Of the intent-to-test samples, 1,074 (98.3%) were included in the final analysis; 1,005 (93.6%) were fresh samples tested between January and May 2019, and 69 (6.4%) were banked (frozen) samples that were later thawed and immediately tested. No difference in assay performance was recognized between fresh and frozen samples. Of the 1,074 samples included in the final analysis, 24.3% (261/1,074) were from individuals less than 18 years of age. Of the 1,074 samples included in the final analysis, 976 (90.9%) had microbes detected by the BIOFIRE BCID2 Panel. All except one of the organism targets on the BIOFIRE BCID2 Panel had a sensitivity of 92.3% or greater, and all but one organism target had a specificity of 96.6% or higher (Table 4). Overall, the sensitivity of the BIOFIRE BCID2 Panel for the detection of on-panel microbes was 98.9% (1,712/1,731). One thousand one hundred twenty organism targets were detected in the 976 positive samples, which included 125 (12.8%) samples that had multiple organism targets detected by the BIOFIRE BCID2 Panel (see Table S1 in the supplemental material).

**TABLE 4:** Organism performance of the BIOFIRE BCID2 Panel

Analyte^*a*^	Sensitivity			Specificity		
	TP/(TP + FN)	%	95% CI (%)	TN/(TN + FP)	%	95% CI (%)
Gram-positive bacteria						
**Enterococcus faecalis**	31/33	93.9	80.4–98.3	1,040/1,041	99.9	99.5–100
**Enterococcus faecium**	27/27	100	87.5–100	1,044/1,047	99.7	99.2–99.9
Listeria monocytogenes	3/3	100	43.9–100	1,071/1,071	100	99.6–100
**Staphylococcus spp.**	471/472	99.8	98.8–100	589/602	97.8	96.3–98.7
Staphylococcus aureus	149/149	100	97.5–100	923/925	99.8	99.2–99.9
**Staphylococcus epidermidis**	221/229	96.5	93.3–98.2	816/845	96.6	95.1–97.6
**Staphylococcus lugdunensis**	4/4	100	51.0–100	1,067/1,070	99.7	99.2–99.9
**Streptococcus spp.**	121/123	98.4	94.3–99.6	949/951	99.8	99.2–99.9
Streptococcus agalactiae	9/9	100	70.1–100	1,065/1,065	100	99.6–100
Streptococcus pneumoniae	26/26	100	87.1–100	1,048/1,048	100	99.6–100
Streptococcus pyogenes	13/14	92.9	68.5–98.7	1,060/1,060	100	99.6–100
Gram-negative bacteria						
**Acinetobacter calcoaceticus*-baumannii* complex**	12/13	92.3	66.7–98.6	1,060/1,061	99.9	99.5–100
**Bacteroides fragilis**	6/6	100	61.0–100	1,065/1,068	99.7	99.2–99.9
***Enterobacterales***	269/270	99.6	97.9–99.9	750/804	93.3	91.3–94.8
Enterobacter cloacae complex	16/16	100	80.6–100	1,058/1,058	100	99.6–100
Escherichia coli	158/159	99.4	96.5–99.9	913/915	99.8	99.2–99.9
**Klebsiella aerogenes**	2/2	100	34.2–100	1,072/1,072	100	99.6–100
Klebsiella oxytoca	8/8	100	67.6–100	1,066/1,066	100	99.6–100
Klebsiella pneumoniae group	55/56	98.2	90.6–99.7	1,018/1,018	100	99.6–100
Proteus spp.	14/14	100	78.5–100	1,059/1,060	99.9	99.5–100
**Salmonella spp.**	5/5	100	56.6–100	1,069/1,069	100	99.6–100
Serratia marcescens	11/11	100	74.1–100	1,063/1,063	100	99.6–100
Haemophilus influenzae	8/8	100	67.6–100	1,066/1,066	100	99.6–100
Neisseria meningitidis	0/0			1,074/1,074	100	99.6–100
Pseudomonas aeruginosa	29/29	100	88.3–100	1,043/1,045	99.8	99.3–99.9
**Stenotrophomonas maltophilia**	7/8	87.5	52.9–97.8	1,066/1,066	100	99.6–100
Yeast						
Candida albicans	12/12	100	75.8–100	1,061/1,062	99.9	99.5–100
**Candida auris**	0/0			1,074/1,074	100	99.6–100
Candida glabrata	10/10	100	72.2–100	1,063/1,064	99.9	99.5–100
Candida krusei	2/2	100	34.2–100	1,072/1,072	100	99.6–100
Candida parapsilosis	8/8	100	67.6–100	1,065/1,066	99.9	99.5–100
Candida tropicalis	5/5	100	56.6–100	1,069/1,069	100	99.6–100
**Cryptococcus neoformans/C. gattii**	0/0			1,074/1,074	100	99.6–100

a Targets novel to the BIOFIRE BCID2 Panel (not included on or modified from the BIOFIRE BCID Panel) are in boldface.

In the 1,074 samples included in the final analysis, 118 off-panel microbes were detected by the comparator (i.e., SoC) method that were not included on the BIOFIRE BCID2 Panel (Table 5). By comparison, 128 organisms detected by the comparator (i.e., SoC) were off panel for the predicate BIOFIRE BCID Panel. Eighty-eight off-panel microbes were present in samples that had no targets detected by the BIOFIRE BCID2 Panel, and 30 off-panel microbes were detected in a total of 30 samples that had additional taxa detected by the BIOFIRE BCID2 Panel.

**TABLE 5:** BIOFIRE BCID2 off-panel microbes identified by SoC^*a*^

Off-panel genus	No. identified (>1)
*Acinetobacter* ^*b*^	2
*Actinomyces*	2
*Aerococcus*	2
*Bacillus*	8
*Bacteroides* ^*b*^	5
*Burkholderia*	2
*Candida* ^*b*^	5
*Clostridium*	8
*Corynebacterium*	11
*Cutibacterium*	9
*Dolosigranulum*	2
*Enterococcus* ^*b*^	4
*Fusobacterium*	3
*Granulicatella*	5
*Micrococcus*	14
*Pseudomonas* ^*b*^	6
*Rothia*	4
*Sphingomonas*	4
*Veillonella*	3
Total	99

a Genera/taxa identified only once include *Abiotrophia*, *Achromobacter*, *Arthrobacter*, *Atopobium*, *Brevibacterium*, *Capnocytophaga*, *Chryseobacterium Eggerthella*, *Finegoldia*, *Globicatella*, *Lactobacillus*, *Lactococcus*, *Moraxella*, *Myroides*, *Paenibacillus*, *Pasteurella*, *Peptoniphilus*, *Peptostreptococcus*, and a coryneform that was not further identified.

b While some members of this genus are on the BIOFIRE BCID2 Panel, those included in this table are not designed to be detected by the panel, including Bacteroides faecis, Bacteroides thetaiotaomicron, Bacteroides vulgatus, Candida dubliniensis, Candida kefyr, Candida lusitaniae, Enterococcus casseliflavus, Enterococcus gallinarum, Pseudomonas guariconensis, Pseudomonas putida, and isolates of the genera Acinetobacter and Pseudomonas that were not further identified.

In the 1,074 samples included in the final analysis, 327 AMR genes were detected and able to be associated with a detected taxon. AMR gene results demonstrated a PPA of 91.2% or higher and an NPA of 97.9% or higher (Table 6). Overall, the sensitivity of detecting on-panel AMR genes was 97.9% (325/332). No detections were observed for IMP, OXA-48-like, or *mcr-1*.

**TABLE 6:** AMR gene performance of the BIOFIRE BCID2 Panel

Analyte (AMR gene)^*a*^	Positive percent agreement			Negative percent agreement		
	TP/(TP + FN)	%	95% CI (%)	TN/(TN + FP)	%	95% CI (%)
**CTX-M**	46/47	97.9	88.9–99.6	312/312	100	98.8–100
**IMP**	0/0			359/359	100	98.9–100
KPC	4/4	100	51.0–100	328/328	100	98.8–100
**NDM**	1/1	100		358/358	100	98.9–100
**OXA-48-like**	0/0			323/323	100	98.8–100
**VIM**	4/4	100	51.0–100	355/355	100	98.9–100
*mecA*/*C*	195/195	100	98.1–100	60/60	100	94.0–100
***mecA*/*C* and MREJ (MRSA)**	52/57	91.2	81.1–96.2	92/94	97.9	92.6–99.4
***mcr-1***	0/0			240/240	100	98.4–100
*vanA*/*B*	23/24	95.8	79.8–99.3	38/38	100	90.8–100

a Targets novel to the BIOFIRE BCID2 Panel (not included on or modified from the BIOFIRE BCID Panel) are in boldface.

Discrepancy analysis, which included but was not limited to additional PCR and sequencing direct from the sample as well as additional identification methods performed on the isolates, was performed for all FN and FP results (Table S2). Of all FN, the BIOFIRE BCID2 Panel result was confirmed for 38%, and the comparator result was confirmed in 42%. Five FN result investigations were inconclusive, meaning that no evidence of the presence of the analyte was found in the sample via additional PCR testing or other investigations. For all FP results, the BIOFIRE BCID2 Panel results were confirmed for 55% and the comparator result was confirmed for 45%. As indicated in the footnote to Table S2, the 45% of FP results in which the comparator results were confirmed were all attributed to the presence of nucleic acid from nonviable Escherichia coli in specific lots of blood culture bottles.

Gram-negative bacilli were recovered from 307 PBC specimens using SoC; at least one *Enterobacterales* isolate was recovered in 87.9% (270/307) of these specimens. Of the 270 specimens from which at least one *Enterobacterales* isolate was recovered, the BIOFIRE BCID2 Panel detected *Enterobacterales* in 269. BIOFIRE BCID2 detected *Enterobacterales* in 54 additional samples in which no isolate was recovered by culture; 53 of these were attributed to the presence of nonviable E. coli in specific lots of blood culture bottles.

Of the 270 samples from which at least one *Enterobacterales* isolate was recovered, 266 had phenotypic BMD results available. Of these 266 samples, ESBL activity was identified phenotypically in 17.3% (46/266), and CTX-M was detected by BIOFIRE BCID2 in 17.3% (46/266) (Table 7). Of the 46 samples with CTX-M detected, three samples did not have phenotypic evidence of an ESBL (two Klebsiella pneumoniae isolates and one Proteus mirabilis isolate). Of the 220 specimens in which CTX-M was undetected by the BIOFIRE BCID2 Panel, ESBL activity was phenotypically absent in 98.6% (217/220) (Table 7); phenotypic ESBL was detected in one K. pneumoniae isolate, one Klebsiella oxytoca isolate, and one P. mirabilis isolate, in which CTX-M was not detected.

**TABLE 7:** BIOFIRE BCID2 Panel CTX-M 2 × 2 performance table^*a*^

BCID2 Panel result	Phenotypic ESBL		BCID2 performance [no. positive/total (%)]
	Yes	No	
Positive	43	3	43/46 (93.5)
Negative	3	217^*b*^	217/220 (98.6)
Total	46	220	

a Four additional specimens had at least one *Enterobacterales* isolate recovered but did not have ESBL phenotypic activity results available.

b The BIOFIRE BCID2 Panel did not detect *Enterobacterales* in one specimen (i.e., the CTX-M result was “NA”), but a Providencia stuartii isolate was recovered that was not phenotypically identified as ESBL positive.

Excluding six samples with detected carbapenemase genes, there were 216 samples in which *Enterobacterales* was detected and CTX-M was undetected by the BIOFIRE BCID2 Panel. Of the 216 samples lacking CTX-M, 95.8% (207/216) tested susceptible and 99.1% (214/216) tested susceptible/SDD to cefepime (Table 8), while 88.0% (190/216) tested susceptible and 91.2% (197/216) tested susceptible/SDD to piperacillin-tazobactam (Table 9). Of the 19 piperacillin-tazobactam resistant isolates, the BIOFIRE BCID2 Panel identified six as common AmpC-producing bacteria, including Enterobacter cloacae complex (5 isolates) and Serratia marcescens (1 isolate). The remaining 13 isolates were identified by the BIOFIRE BCID2 Panel as E. coli (4 isolates), K. pneumoniae (4 isolates), K. oxytoca (1 isolates), Proteus sp. (2 isolates) (final identifications were P. mirabilis), and *Enterobacterales* without a more specific identification (2 isolates) (final identifications of Hafnia alvei and Citrobacter freundii).

**TABLE 8:** BIOFIRE BCID2 Panel CTX-M results compared to cefepime susceptibility^*a*^

BCID2 Panel CTX-M result	Cefepime susceptibility		
	R	SDD	S
Positive	36	6	2
Negative	2	7	207
Total	38	13	209

a The MICs were determined using broth microdilution and interpreted using the CLSI standards (6) as susceptible (S), susceptible—dose dependent (SDD), or resistant (R). Six specimens with KPC, VIM, and/or NDM were excluded from this analysis (*n* = 260).

**TABLE 9:** BIOFIRE BCID2 Panel CTX-M results compared to piperacillin-tazobactam susceptibility^*a*^

BCID2 Panel CTX-M result	Piperacillin-tazobactam susceptibility		
	R	SDD	S
Positive	13	3	28
Negative	19	7	190
Total	32	10	218

a The MICs were determined using broth microdilution and interpreted using the CLSI standards (6) as susceptible (S), susceptible—dose dependent (SDD), or resistant (R). Six specimens with KPC, VIM, and/or NDM were excluded from this analysis (*n* = 260).

Of the 270 samples from which at least one *Enterobacterales* isolate was recovered, 266 samples had BMD susceptibility results for meropenem (Table 10). Carbapenem resistance was identified phenotypically in 2.3% (6/266) of the samples, and a carbapenemase gene was detected by the BIOFIRE BCID2 Panel in 2.3% (6/266). Of the carbapenem resistance gene markers, KPC was detected in 1.5% (4/266), VIM was detected in 0.4% (1/266), and NDM with VIM was detected in 0.4% (1/266). Of the 260 samples in which the BIOFIRE BCID2 Panel did not detect a carbapenemase gene, 99.6% (259/260) tested phenotypically susceptible to meropenem. Of the samples with meropenem-susceptible isolates, 99.2% (257/259) had MICs of ≤0.12 μg/mL.

**TABLE 10:** BIOFIRE BCID2 Panel carbapenem resistance gene 2 × 2 performance table^*a*^

BCID2 Panel result (any gene)	Phenotypic carbapenem resistance		BCID2 performance [no. positive/total (%)]
	R	S	
Positive	5^*b*^	1^*c*^	5/6 (83.3)
Negative	1	259	259/260 (99.6)
Total	6	260	

a Four additional specimens had at least one *Enterobacterales* isolate recovered but did not have phenotypic susceptibility results available.

b KPC was detected in four specimens; NDM and VIM were detected in one specimen.

c VIM was detected in one specimen.

In summary, the BIOFIRE BCID2 Panel demonstrated an overall sensitivity of 98.9% (1,712/1,731) and an overall specificity of 99.6% (33,592/33,711) for Gram-positive bacteria, Gram-negative bacteria, and yeast targets compared to SoC from PBC specimens. The BIOFIRE BCID2 Panel also demonstrated an overall PPA of 97.9% (325/332) and an overall NPA of 99.9% (2,465/2,467) for AMR genes. The presence or absence of AMR genes in *Enterobacterales* correlated closely with phenotypic susceptibility and resistance.

## DISCUSSION

The BIOFIRE BCID2 Panel is differentiated from the original BIOFIRE BCID Panel in that it has increased specificity of some taxonomic identifications, additional identifiable taxa, and additional genetic resistance markers. Notably, the BIOFIRE BCID2 Panel has the following modifications from the BIOFIRE BCID Panel: expanded detection of the Acinetobacter calcoaceticus-Acinetobacter baumannii complex, *Enterobacterales*, Staphylococcus spp., and Streptococcus spp.; individual species/genus assays instead of higher-level interpretations for Enterococcus faecalis, Enterococcus faecium, Klebsiella aerogenes, Salmonella spp., Staphylococcus epidermidis, and Staphylococcus lugdunensis; and the addition of Bacteroides fragilis, Candida auris, Cryptococcus neoformans/Cryptococcus gattii, and Stenotrophomonas maltophilia. Further, the AMR gene menu on the BIOFIRE BCID2 Panel has been expanded to include targets for CTX-M, IMP, NDM, OXA-48-like, VIM, *mecA*/*C*, and MREJ (MRSA), and *mcr-1*, which can help to guide antimicrobial selection before phenotypic antimicrobial susceptibility testing (AST) results and interpretations are available.

Although C. auris and Cryptococcus neoformans/Cryptococcus gattii fungemia is rarely encountered and was not present in any samples from this clinical study, the performance of the panel for these pathogens was evaluated using archived and seeded PBC samples, and these analytes are included in the FDA-cleared assay. Rapid identification of these yeasts should provide clinical value due to their resistance to antifungal agents. B. fragilis group and S. maltophilia were identified by the BIOFIRE BCID2 Panel in 100% (6/6) and 87.5% (7/8) of the samples containing these bacteria in the current study (Table 4). Rapid identification of S. maltophilia is important because of its resistance to commonly used empirical Gram-negative antibiotics (8).

Potentially the most clinically valuable addition to the BIOFIRE BCID2 Panel is the CTX-M target, which was identified in 17% of samples containing *Enterobacterales*. The presence of CTX-M correlated closely with resistance to cefepime (Table 8), and piperacillin-tazobactam is often avoided when an ESBL like CTX-M is present because of the findings from the MERINO trial (9). *Enterobacterales* were reliably susceptible to meropenem when carbapenemase genes were not detected (Table 10). Similar to the findings of a study by Spafford and colleagues, genetic resistance markers like CTX-M and KPC are able to identify many *Enterobacterales* isolates that lack susceptibility to relevant beta-lactams, but a substantial minority of *Enterobacterales* isolates that are not susceptible to third- or fourth-generation cephalosporins do not have CTX-M (10). When the presence or absence of a single gene marker (e.g., CTX-M) is used to guide empirical antimicrobial therapy, it is important to consider not only the sensitivity of an assay but also its negative predictive value, which is dependent upon local prevalence of overall resistance and also resistance mechanisms (11). In the future, characterization and analysis of full genomic data rather than single gene markers could be used to more accurately predict phenotypic susceptibility and resistance (12). With the addition of the expanded *Enterobacterales* AMR targets, local laboratories in collaboration with local antimicrobial stewardship programs will be able to design optimized empirical antibiotic regimens that can be informed by the BIOFIRE BCID2 Panel AMR results.

A limitation of the BIOFIRE BCID2 Panel revealed by the study is the ability of the assay to detect nucleic acid from nonviable *Enterobacterales* (Table 4 and Table S2). In practice, this limitation can be potentially mitigated by requiring Gram stain findings to be congruent with an “*Enterobacterales*” identification when no specific taxa within *Enterobacterales* are identified by the BIOFIRE BCID2 Panel. This mitigation strategy would be in line with the IFU, which states that BIOFIRE BCID2 Panel results are intended to be interpreted in conjunction with Gram stain results.

The greatest limitation of the study is that not all targets were thoroughly interrogated, as some targets are rarely encountered in clinical practice. This is a common challenge in clinical studies that evaluate broad, multitarget panels. This limitation is mitigated by evaluating the performance of the test with contrived samples. The performance of the BIOFIRE BCID2 Panel with contrived samples was performed, and the results can be found in the BIOFIRE BCID2 Panel IFU (https://www.online-ifu.com/ITI0048).

We conclude that the BIOFIRE BCID2 Panel will be a valuable addition to the growing repertoire of IVD tools to rapidly identify and characterize microbes recovered by blood culture.
