# Supplementary material for: Multicenter Evaluation of the BIOFIRE Blood Culture Identification 2 Panel for Detection of Bacteria, Yeasts, and Antimicrobial Resistance Genes in Positive Blood Culture Samples
Source: J Clin Microbiol. 2023 May 25;61(6):e01891-22. doi: 10.1128/jcm.01891-22 (PMC10281132; doi:10.1128/jcm.01891-22)
Supplement: Supplemental file 1 — Supplemental material. Download jcm.01891-22-s0001.docx, DOCX file, 0.03 MB [file jcm.01891-22-s0001.docx]

Supplementary Table 1. Prevalence of Microbial Targets in Co-Detections as Determined by the BIOFIRE^®^ BCID2 Panel

| **Organism** | **Prevalence in Co-Detections (N=125)** |
| --- | --- |
|  |  |
| **Gram-Positive Bacteria** | |
| *Enterococcus faecalis* | 10 |
| *Enterococcus faecium* | 13 |
| *Listeria monocytogenes* | 1 |
| *Staphylococcus* spp. | 80 |
| *Staphylococcus aureus* | 18 |
| *Staphylococcus epidermidis* | 53 |
| *Staphylococcus lugdunensis* | 3 |
| *Streptococcus* spp. | 32 |
| *Streptococcus agalactiae* | 2 |
| *Streptococcus pneumoniae* | 7 |
| *Streptococcus pyogenes* | 0 |
| **Gram-Negative Bacteria** | |
| *Acinetobacter calcoaceticus-baumannii* complex | 5 |
| *Bacteroides fragilis* | 3 |
| *Enterobacterales* | 80 |
| *Enterobacter cloacae* complex | 3 |
| *Escherichia coli* | 18 |
| *Klebsiella aerogenes* | 1 |
| *Klebsiella oxytoca* | 3 |
| *Klebsiella pneumoniae* group | 11 |
| *Proteus* spp. | 4 |
| *Salmonella* spp. | 0 |
| *Serratia marcescens* | 0 |
| *Haemophilus influenzae* | 3 |
| *Neisseria meningitidis* | 0 |
| *Pseudomonas aeruginosa* | 9 |
| *Stenotrophomonas maltophilia* | 0 |
| **Yeast** | |
| *Candida albicans* | 4 |
| *Candida auris* | 0 |
| *Candida glabrata* | 3 |
| *Candida krusei* | 2 |
| *Candida parapsilosis* | 3 |
| *Candida tropicalis* | 4 |
| *Cryptococcus neoformans/gattii* | 0 |

Supplementary Table 2. Summary of outcomes of discrepant results investigation

| Result and Analyte | | No. of Results | No. of investigations | | |
| --- | --- | --- | --- | --- | --- |
|  |  |  | Comparator result confirmed | BIOFIRE^®^ BCID2 result confirmed | Inconclusive |
| BIOFIRE^®^ BCID2 Panel FN | |  |  |  |  |
|  | *Enterococcus faecalis* | 2 | 2 | 0 | 0 |
|  | *Staphylococcus* spp. | 1 | 0 | 0 | 1 |
|  | *Staphylococcus epidermidis* | 8 | 3 | 5 | 0 |
|  | *Streptococcus* spp. | 2 | 1 | 0 | 1 |
|  | *Streptococcus pyogenes* (Group A) | 1 | 1 | 0 | 0 |
|  | ACB complex | 1 | 1 | 0 | 0 |
|  | *Enterobacterales* | 1 | 0 | 0 | 1 |
|  | *Escherichia coli* | 1 | 0 | 0 | 1 |
|  | *Klebsiella pneumoniae* group | 1 | 1 | 0 | 0 |
|  | *Stenotrophomonas maltophilia* | 1 | 1 | 0 | 0 |
|  | CTX-M | 1 | 0 | 0 | 1 |
|  | *mecA/C* and MREJ (MRSA) | 5 | 0 | 5 | 0 |
|  | *vanA/B* | 1 | 1 | 0 | 0 |
|  |  |  |  |  |  |
|  | Total | 26 | 11 | 10 | 5 |
|  | % of total FN results |  | 42% | 38% | 19% |
|  |  |  |  |  |  |
| BIOFIRE^®^ BCID2 Panel FP | |  |  |  |  |
|  | *Enterococcus faecalis* | 1 | 0 | 1 | 0 |
|  | *Enterococcus faecium* | 3 | 0 | 3 | 0 |
|  | *Staphylococcus* spp. | 13 | 0 | 13 | 0 |
|  | *Staphylococcus aureus* | 2 | 0 | 2 | 0 |
|  | *Staphylococcus epidermidis* | 29 | 0 | 29 | 0 |
|  | *Staphylococcus lugdunensis* | 3 | 0 | 3 | 0 |
|  | *Streptococcus* spp. | 2 | 0 | 2 | 0 |
|  | ACB complex | 1 | 0 | 1 | 0 |
|  | *Bacteroides fragilis* | 3 | 0 | 3 | 0 |
|  | *Enterobacterales* | 54 | 53^a^ | 1 | 0 |
|  | *Escherichia coli* | 2 | 2^a^ | 0 | 0 |
|  | *Proteus* spp. | 1 | 0 | 1 | 0 |
|  | *Pseudomonas aeruginosa* | 2 | 0 | 2 | 0 |
|  | *Candida albicans* | 1 | 0 | 1 | 0 |
|  | *Candida glabrata* | 1 | 0 | 1 | 0 |
|  | *Candida parapsilosis* | 1 | 0 | 1 | 0 |
|  | *mecA/C* and MREJ (MRSA) | 2 | 0 | 2 | 0 |
|  |  |  |  |  |  |
|  | Total | 121 | 55 | 66 | 0 |
|  | % of total FP results |  | 45% | 55% | 0% |

^a^ Fifty-three (53) of 54 FP *Enterobacterales* results and both FP *E. coli* results were attributed to the presence of nucleic acid from non-viable *E. coli* in specific lots of blood culture bottles
